# Predictive Utility of the Glasgow Coma Scale and the Head Abbreviated Injury Scale in Traumatic Brain Injury: Results from the National Trauma Registry of Iran

**DOI:** 10.34172/aim.2022.81

**Published:** 2022-08-01

**Authors:** Moein Khormali, Pooya Payandemehr, Sahar Zafarmandi, Vali Baigi, Mohammadreza Zafarghand, Mahdi Sharif-Alhoseini

**Affiliations:** ^1^Sina Trauma and Surgery Research Center, Tehran University of Medical Sciences, Tehran, Iran; ^2^Department of Emergency Medicine, Sina Hospital, Tehran University of Medical Sciences, Tehran, Iran

**Keywords:** Abbreviated Injury Scale, Glasgow Coma Scale, Outcome measures, Trauma severity indices, Traumatic brain injury

## Abstract

**Background:**

Trauma severity indices are commonly used to describe the severity of sustained injuries in a quantitative manner perceivable by healthcare providers in different settings. In this study, we aimed to assess the predictive utility of the Glasgow Coma Scale (GCS) and the 2015 revision of the head Abbreviated Injury Scale (head AIS) as two of the most widely used severity indices for traumatic brain injury (TBI).

**Methods:**

In this cross-sectional study, we used data from the National Trauma Registry of Iran. The area under the receiver operating characteristic curve (AUROC) was calculated to assess the utility of GCS and head AIS scores in predicting patients’ outcomes.

**Results:**

A total of 321 patients, predominantly males (81.9%) with an average age of 41.9 (±19.5) years were enrolled in the study. The most common cause of injury was road traffic accidents (73.5%) followed by falls (20.2%). The mean admission GCS and head AIS scores were 13.5 (±3.2) and 2.5 (±1.0), respectively. AUROC of the GCS was significantly higher than the head AIS for all outcome variables (*P*<0.05). AUROC of both severity scoring systems for predicting in-hospital mortality was significantly higher in the 15–44 age group than the 65 or older age group (*P*<0.05).

**Conclusion:**

Based on our study results, GCS had better performance in predicting patients’ outcomes than the head AIS. Also, we found that age significantly affected the ability of these indices in predicting in-hospital mortality of TBI patients.

## Introduction

 Traumatic brain injury (TBI) is a devastating condition estimated to have an annual worldwide incidence and prevalence of 27.08 and 55.50 million cases, respectively.^1^ The incidence rates of TBI have increased over time.^1^ In high-income countries, the increasing fall episodes resulting in TBI, especially in the elderly population, are blamed, whereas in low and middle-income countries, the increasing number of road traffic accidents may be considered the cause.^1-3^ Due to the high mortality and morbidity of TBI and its financial and non-financial impacts on societies, it must be considered a high priority issue for health systems which requires global attention.

 Trauma severity indices are commonly used to describe the severity of sustained injuries in a quantitative manner perceivable by healthcare providers in different settings.^4,5^ These indices can be used to predict patients’ outcomes and thus may help healthcare providers in timely clinical decision-making. Physiologic indices assess the impact of injuries on patients’ functional reserves, whereas anatomic indices are based on a detailed description of received injuries.^6^

 The Glasgow Coma Scale (GCS), a physiologic score developed in 1974, is one of the most commonly used scoring systems.^7^ It has some limitations. For instance, in patients with intoxication or metabolic abnormality or those who have been sedated or received muscle relaxants, and patients with endotracheal tubes or injury around their eyes that may interfere with the eye-opening response, it may not be an accurate prognostic indicator.^5,8^ Also, some problems regarding inter-rater reliability of GCS have been reported in previous studies.^9,10^

 The Abbreviated Injury Scale (AIS) developed by the Association for the Advancement of Automotive Medicine, is an anatomic score that takes into account all body regions, including the head, and the severity of injury in each body region ranges from 1 (minor injury) to 6 (unsurvivable injury).^11,12^ Considering the injured anatomical structures (such as blood vessels, nerves, internal organs, bones, and joints), nature, and level of injury, a 7-digit code is generated that consists of a 6-digit pre-dot and a one-digit post-dote code. The post-dot code indicates injury severity. The AIS also has some limitations. For instance, because of the anatomic nature and the need for a detailed description of received injuries, calculation of the AIS score takes a longer time and it is less feasible in emergency settings.^6^ The AIS 2015 revision is the latest version of this injury coding dictionary.^11^ To enhance AIS coding accuracy, there have been some changes in the 2015 revision, including its head chapter, compared to earlier versions. For instance, some changes have been made in concussion and diffuse axonal injury coding and differentiation of coma and loss of consciousness based on consultation with neurotrauma specialists.^11^

 Due to the widespread use of AIS in injury severity scoring, we aimed to assess and compare the utility of GCS and the latest revision of head AIS in TBI outcome prediction.

## Patients and Methods

 In this cross-sectional study, we used data from the National Trauma Registry of Iran (NTRI). NTRI is a hospital-based registry conducted in some of the major trauma centers across the country with the support of Iran’s Ministry of Health and Medical Education. The registry process, minimum dataset, and data quality control in the NTRI have been previously described.^13,14^ Patients who meet one of the NTRI inclusion criteria (hospital length of stay [LOS] more than 24 hours, death after injury, or transfer from intensive care units [ICUs] of other hospitals) are included in the registry. Patients’ data are extracted from medical profiles, hospital information systems (HIS), and face-to-face interviews by at least two trained nurses in each collaborating center. A trained physician then controls data in terms of completeness, accuracy, and consistency. After quality review and verification, the data are stored in the NTRI databank.

 All patients aged 15 years or older with head trauma (head AIS severity of 1 to 6) registered in the NTRI whose hospital admission dates were from January 1 to December 31, 2019, were included in this study. Patients with unknown head AIS severity score, patients transported from NTRI collaborating centers to other hospitals to continue the treatment process, patients who left the hospital with personal consent or without physician discharge, patients with unknown hospital discharge status, patients with penetrating injuries (gunshot or stab wound), and those with major extracranial injuries were excluded from the study. Major extracranial injuries were defined as injuries sustained to body regions other than the head with AIS severity greater than 3 (AIS > 3).

 Data regarding baseline, injury, and admission characteristics, as well as outcome and predictor (the GCS and AIS scores) variables were obtained from the NTRI databank. Outcomes assessed in this study were in-hospital mortality, ICU admission, ICU LOS for two days or more, and prolonged ICU stay (ICU LOS 14 days or more). Total GCS scores were categorized as severe (score of 8 or less), moderate (score of 9-12), and mild TBI (score of 13-15). If a patient had more than one head AIS injury code, the highest severity score was used for analysis as the patient’s head AIS score.

 Comparisons of the GCS and the head AIS scores between outcome subgroups were made with the Mann–Whitney U non-parametric method. Statistical analyses were performed using the IBM SPSS Statistics, version 22 (IBM Corp., Armonk, NY, USA). The area under the receiver operating characteristic curve (AUROC) was also calculated to assess the utility of the GCS and head AIS scores in predicting patients’ outcomes. MedCalc software trial version 18.11 was used to calculate and compare AUROC between study subgroups (MedCalc Software, Ostend, Belgium). Associations with a *P* < 0.05 were considered statistically significant.

## Results

 On the basis of the inclusion and exclusion criteria, 321 patients were enrolled in the study. The mean (standard deviation) and median (interquartile range) age were 41.9 (19.5) and 37 (29.5) years, respectively. The patients’ age ranged from 15 to 91 years. The mean (standard deviation) GCS and head AIS scores were 13.5 (3.2) and 2.5 (1.0), respectively. Baseline and injury characteristics, descriptive statistics of predictor and outcome variables, and distribution of predictor variables in outcome subgroups are shown in Tables 1, 2 and Figures 1, 2. The most common cause of injury was road traffic accidents (n = 236 [73.5 %]) followed by falls (n = 65 [20.2 %]). Among road traffic accident victims, the majority of patients were motorcyclists (n = 127 [53.8 %]) followed by car occupants (n = 58 [24.6 %]) and pedestrians (n = 40 [16.9 %]). In the 15–44 years and 45–64 years age groups, most injuries were due to road traffic accidents, while in the 65 years and older age group, falls were the most common cause of injury (Figure 1). Among fall victims, 30 (46.1 %) patients fell from standing height, 25 (38.5 %) patients fell from a height lower than 3 meters, and 10 (15.4 %) fell from a height of 3 meters or higher. The median and interquartile range of predictors in outcome subgroups are shown in Table 2. There were significant differences between outcome subgroups in terms of predictor variables distribution (*P* < 0.001). AUROCs of predictor variables for different outcomes are shown in Table 3. Observed differences between the GCS and head AIS in AUROCs were statistically significant for all four outcome variables (Table 3).

**Table 1:** Characteristics of Patients with Traumatic Brain Injury (N = 321)

	**Frequency (%)**
Gender	
Male	263 (81.9)
Female	58 (18.1)
Age group (y)	
15–44	194 (60.4)
45–64	78 (24.3)
≥ 65	49 (15.3)
Cause of injury	
Road traffic accident	236 (73.5)
Fall	65 (20.3)
Other blunt trauma	20 (6.2)
Education level	
Illiterate	54 (16.8)
Non-university degree	229 (71.3)
University degree	25 (7.8)
Unknown	13 (4.1)
Nationality	
Iranian	297 (92.5)
Non-Iranian	24 (7.5)

**Table 2:** Predictor Variables in Outcome Subgroups

	**Frequency (%)**	GCS^*^ Median (IQR)	***P *** **Value**	**Head AIS Median (IQR)**	***P *** **Value**
Discharge status					
Dead	23 (7.2)	8.5 (10)	< 0.001	3 (0)	< 0.001
Alive	298 (92.8)	15 (0)		2 (1)	
ICU admission					
Yes	109 (34.0)	14 (8)	< 0.001	3 (1)	< 0.001
No	212 (66.0)	15 (0)		2 (2)	
ICU LOS ≥ 2 days					
Yes	89 (27.7)	12.5 (9)	< 0.001	3 (1)	< 0.001
No	232 (72.3)	15 (0)		2 (1)	
ICU LOS ≥ 14 days					
Yes	29 (9.0)	9 (9)	< 0.001	3 (1)	< 0.001
No	292 (91.0)	15 (0)		2.5 (1)	

* 8 patients had unknown GCS scores. IQR, Interquartile range; ICU, Intensive care unit; LOS, Length of stay; GCS, Glasgow Coma Scale; AIS, Abbreviated Injury Scale.

**Figure 1:**
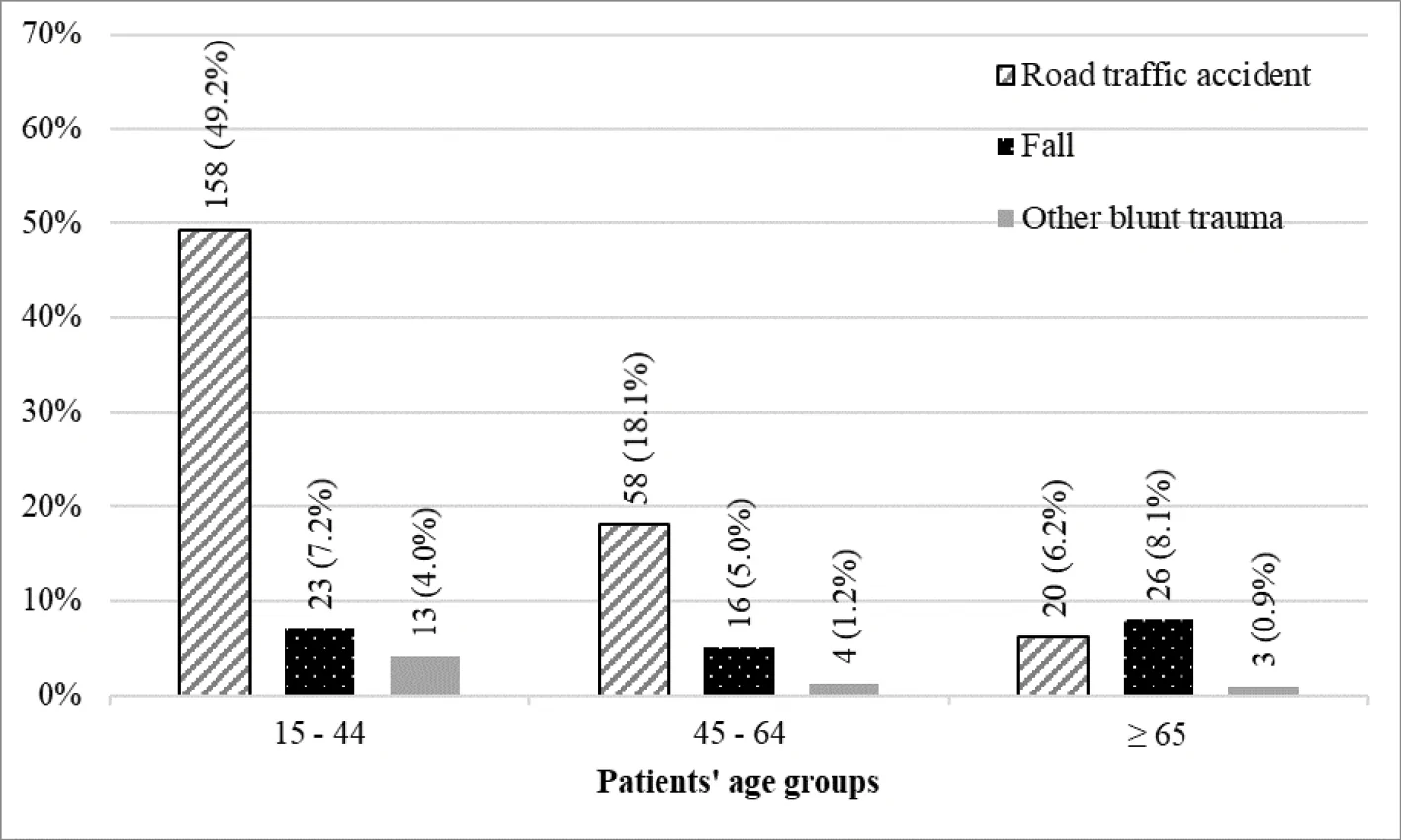
Cause of Injury in Age Groups

**Figure 2:**
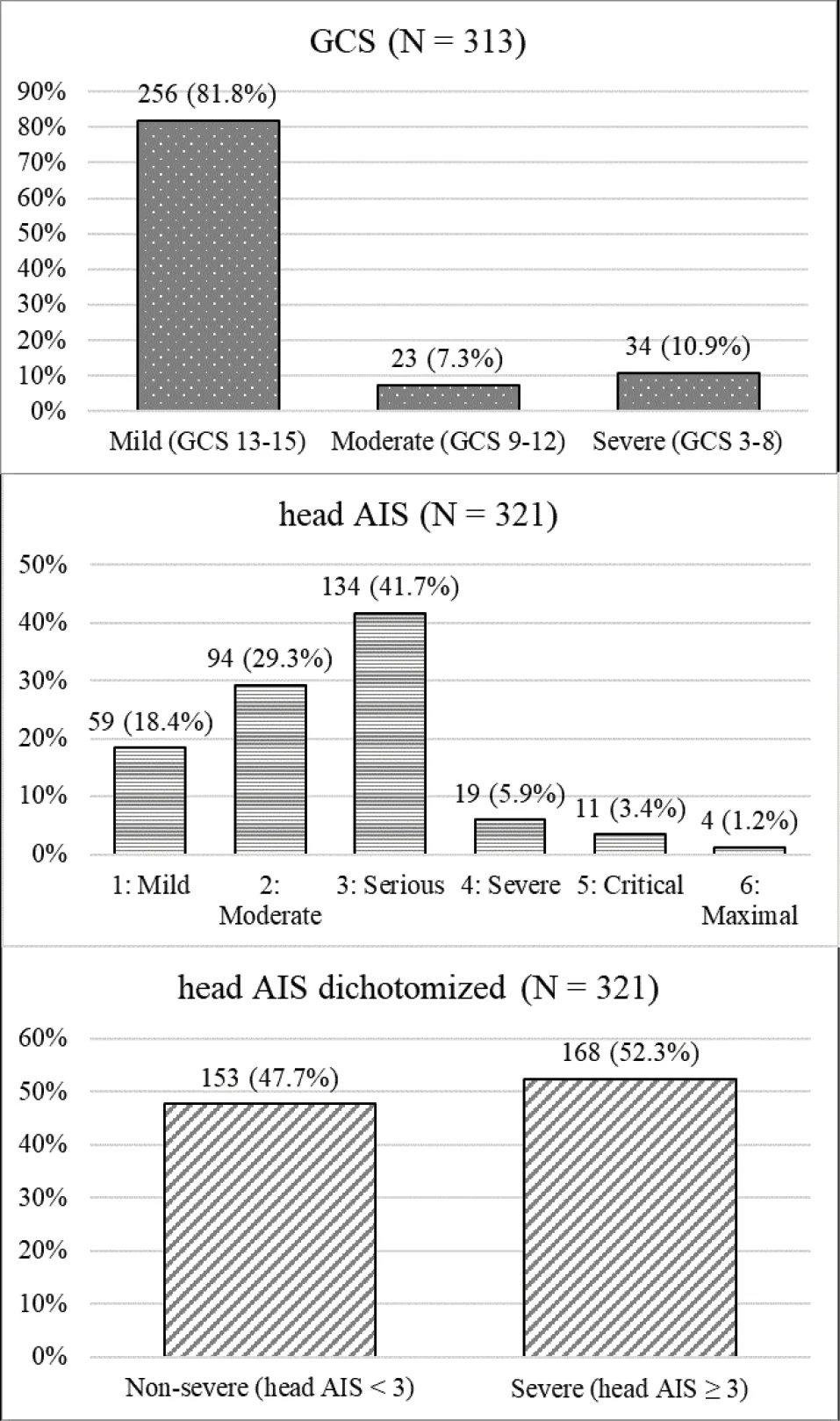
Distribution of TBI Severity Based on GCS* Category, Head AIS, and Dichotomized Head AIS *8 patients had unknown GCS scores. GCS, Glasgow Coma Scale; AIS, Abbreviated Injury Scale

**Table 3:** AUROC of GCS and Head AIS for Outcome Prediction

	**AUROC (95 % Confidence Interval)**		**Comparison of AUROC between the GCS and head AIS scores**
	**GCS**	**Head AIS**	
Discharge status	0.837 (0.741–0.933) *P* < 0.001	0.718 (0.647–0.788) *P* < 0.001	*P* = 0.021
ICU admission	0.759 (0.706–0.811) *P* < 0.001	0.681 (0.625–0.737) *P* < 0.001	*P* = 0.032
ICU LOS ≥ 2 days	0.798 (0.743–0.853) *P* < 0.001	0.685 (0.628–0.743) *P*< 0.001	*P* = 0.003
ICU LOS ≥ 14 days	0.875 (0.813–0.936) *P* < 0.001	0.693 (0.612–0.775) *P* < 0.001	*P*< 0.001

AOROC, Area under the receiver operating characteristic curve; ICU, Intensive care unit; LOS, Length of stay; GCS, Glasgow Coma Scale; AIS, Abbreviated Injury Scale.

 In order to assess the effect of age on the predictive utility of GCS and head AIS scores, we calculated the AUROC for age subgroups (Table 4). Although the AUROC was higher in the 15–44 age group than the 45-64 age group and it was also higher in the 15–44 and 45–64 age groups than patients with 65 years of age or older, the observed difference was only significant between the 15–44 and 65 years or older age groups (*P*= 0.0496 for GCS and *P* = 0.023 for head AIS).

**Table 4:** AUROC of GCS and Head AIS for Predicting In-hospital Mortality in Age Subgroups

	**Patients’ Status at Hospital Discharge (N=321)**		**AUROC (95 % Confidence Interval)**	
	**Dead**	**Alive**	GCS^*^	**Head AIS**
15–44	7 (3.6 %)	187 (96.4 %)	0.934 (0.876–0.991) *P* < 0.001	0.824 (0.717–0.931) *P* < 0.001
45–64	7 (9.0 %)	71 (91.0 %)	0.875 (0.714–1.000) *P* < 0.001	0.704 (0.627–0.782) *P* < 0.001
≥ 65	9 (18.4 %)	40 (81.6 %)	0.705 (0.484–0.926) *P* = 0.070	0.596 (0.431–0.760) *P* = 0.253

* 8 patients had unknown GCS scores. AUROC, Area under the receiver operating characteristic curve; GCS, Glasgow Coma Scale; AIS, Abbreviated Injury Scale.

## Discussion

 In our study, the AUROC of GCS for predicting mortality was 0.837. In a study by Gill et al on more than 8000 trauma patients in a level 1 trauma center registry, AUROC was reported to be 0.906.^15^ The median (IQR) patients’ age in their study was 24 (15–38) years.^15^ In a study by Settervall et al on 277 patients with blunt TBI, the predictive ability of GCS score after initial care was reported to be moderate with AUROC of 0.747.^16^ In the mentioned study, the patients’ age ranged from 14 to 92 with a mean (standard deviation) of 37.7 (16.6) years. Settervall et al concluded that GCS could be used to predict the in-hospital mortality of patients with TBI, but it should be noted that its predictive ability is moderate.^16^ Joosse et al conducted a study on 49 patients with TBI and reported the AUROC of GCS for predicting mortality to be 0.756.^17^ We hypothesized that one of the reasons for these varying AUROC values reported in previous studies could be the different distribution of age groups in study participants. Kehoe et al showed that elderly patients with TBI had a higher presenting GCS score than younger patients despite having a similar AIS severity score.^18^ Also, elderly deceased patients with TBI had higher presenting GCS scores than younger deceased patients with TBI.^18^ Kehoe et al concluded that it is necessary to reconsider and modify the GCS score as a triage tool for elderly patients with a head injury. So, the higher AUROC in the study by Gill et al compared with findings of our study and also by Settervall et al may be at least partly justified by the different age distribution of study participants. Demetriades et al also reported that the GCS and head AIS prognostic values were affected by patients’ age.^19^ In other words, patients of different age but with similar GCS or similar head AIS scores can have different survival outcomes.^19^ Our results also support these findings, since the predictive ability of the GCS and head AIS for mortality was higher in the 15–44 age group than the 45–64 age group and it was also higher in the 15–44 and 45–64 age groups than patients with 65 years of age or older. However, only the difference of AUROC between the 15–44 and 65 or older age groups was statistically significant (borderline significant for the GCS score). We could not perform further subgroup analyses due to the small number of registered death outcomes in our study sample.

 Different findings have been reported regarding GCS and head AIS performance comparison in predicting TBI outcome. While some studies reported higher performance for the head AIS as an anatomic score, other studies reported higher performance for the GCS score.^20,21^ Based on our study results, AUROC of the head AIS in predicting the outcome of patients with TBI was lower than GCS. Although AIS is a commonly used tool for severity score calculation and outcome prediction in trauma patients worldwide, it is not routinely collected and documented in Iranian hospitals. The NTRI determines AIS severity codes in a retrospective manner based on diagnosis documented in the HIS according to the 10th revision of the International Classification of Diseases (ICD-10) and patients’ profiles. Since documented ICD-10 diagnosis codes may be less specific with fewer details, exact conversion and determination of severity codes may not be possible. Thus, the validity of the AIS severity codes may be lower than that of direct coding. For instance, according to the AIS dictionary, cerebral epidural hemorrhage with unknown hemorrhage volume (epidural not further specified; epidural NFS) takes the severity score of 3, but if its volume is calculated on clinical images, it can take a severity score of 2, 4, or even 5.^11^ So, a possible explanation for differences in predictive ability of the head AIS compared with the GCS in previous studies may be that these studies have coded severities with varying degrees of specificity and details. Airaksinen et al also recommended that more specific ICD-10 codes can make the ICD-10 to AIS codes conversion more reliable.^22^ It should be noted that some tools have been developed to enhance the validity of converting ICD-10 codes to AIS severity codes in high-scale population-based injury research or circumstances where direct coding is less feasible.^23-25^ However, none of them are based on the 2015 revision of the AIS dictionary to the best of our knowledge.

 Our study had some limitations. We extracted the GCS scores at a single time point and did not consider the GCS score changes during the hospitalization period. Also, scores used in this study were extracted in a retrospective manner from patients’ profiles and hospital information systems. So, we were mostly dependent on previously documented data whose accuracy and reliability could not be determined. Another limitation of our study was the low proportion of death outcomes in the study sample. Foreman et al defined mild injury as a GCS score of 13 to 15 with an abnormal computed tomographic (CT) scan.^21^ However, we did not consider patients’ brain CT findings in our study and defined mild injury as a GCS score of 13–15. Also, the NTRI is a hospital-based registry and does not register deaths occurring outside hospital. These can be the reasons for the low proportion of death outcomes in our study sample. Another limitation of our study was that we only measured patients’ outcomes as alive or dead at hospital discharge, and post-discharge follow-up was not performed.

 In future studies, as well as reporting the AIS severity scores, it should also be reported how detailed the AIS codes were calculated. In the AIS dictionary, it has been recommended that there should be two severity scoring systems, one for databases with detailed injury descriptions and another for databases with fewer-detailed and more general injury descriptions.^11^ Instead of having two separate scoring systems, an index can be developed for reporting the extent of AIS coding detail. For instance, in order for the AIS codes from different databases with different injury description details to be comparable, the percentage of AIS codes in each database ending in NFS can be reported. Also, we recommend that future studies from the NTRI cover more extended periods with higher sample sizes in order to facilitate further subgroup analyses, and if possible, more exact age thresholds for predictive utility changes of the GCS and head AIS be determined.

 Based on our study results, the GCS had better performance in predicting patients’ outcomes than the head AIS. Also, we found that age significantly affected the ability of GCS and head AIS in predicting the in-hospital mortality of patients with TBI. Despite the limitations of our study and given the insufficiency of studies and reliable data on outcomes of patients with TBI in developing countries, we believe that the findings of this study can be the basis for future large-scale studies in Iran.

## References

[1] GBD 2016 Traumatic Brain Injury and Spinal Cord Injury Collaborators. Global, regional, and national burden of traumatic brain injury and spinal cord injury, 1990-2016: a systematic analysis for the Global Burden of Disease Study 2016. Lancet Neurol. 2019;18(1):56-87. 10.1016/s1474-4422(18)30415-0 PMC6291456 30497965

[2] Maas AIR, Menon DK, Adelson PD, Andelic N, Bell MJ, Belli A (2017). Traumatic brain injury: integrated approaches to improve prevention, clinical care, and research. Lancet Neurol.

[3] Kleiven S, Peloso PM, von Holst H (2003). The epidemiology of head injuries in Sweden from 1987 to 2000. Inj Control Saf Promot.

[4] Chawda MN, Hildebrand F, Pape HC, Giannoudis PV (2004). Predicting outcome after multiple trauma: which scoring system?. Injury.

[5] Senkowski CK, McKenney MG (1999). Trauma scoring systems: a review. J Am Coll Surg.

[6] Kingston R, O’Flanagan SJ (2000). Scoring systems in trauma. Ir J Med Sci.

[7] Teasdale G, Jennett B (1974). Assessment of coma and impaired consciousness A practical scale. Lancet.

[8] Stocchetti N, Pagan F, Calappi E, Canavesi K, Beretta L, Citerio G (2004). Inaccurate early assessment of neurological severity in head injury. J Neurotrauma.

[9] Zuercher M, Ummenhofer W, Baltussen A, Walder B (2009). The use of Glasgow Coma Scale in injury assessment: a critical review. Brain Inj.

[10] Gill MR, Reiley DG, Green SM (2004). Interrater reliability of Glasgow Coma Scale scores in the emergency department. Ann Emerg Med.

[11] Abbreviated Injury Scale: 2015 Revision (6 ed.) Chicago, IL: Association for the Advancement of Automotive Medicine; 2015.

[12] Rating the severity of tissue damage. I. The abbreviated scale. JAMA 1971;215(2):277-80. 10.1001/jama.1971.03180150059012. 5107365

[13] Sharif-Alhoseini M, Zafarghandi M, Rahimi-Movaghar V, Heidari Z, Naghdi K, Bahrami S (2019). National Trauma Registry of Iran: A Pilot Phase at a Major Trauma Center in Tehran. Arch Iran Med.

[14] Ghodsi Z, Rahimi Movaghar V, Zafarghandi M, Saadat S, Mohammadzadeh M, Fazel M (2017). The Minimum Dataset and Inclusion Criteria for the National Trauma Registry of Iran: A Qualitative Study. Arch Trauma Res.

[15] Gill M, Windemuth R, Steele R, Green SM (2005). A comparison of the Glasgow Coma Scale score to simplified alternative scores for the prediction of traumatic brain injury outcomes. Ann Emerg Med.

[16] Settervall CH, de Sousa RM, Fürbringer e Silva SC (2011). In-hospital mortality and the Glasgow Coma Scale in the first 72 hours after traumatic brain injury. Rev Lat Am Enfermagem.

[17] Joosse P, Smit G, Arendshorst RJ, Soedarmo S, Ponsen KJ, Goslings JC (2009). Outcome and prognostic factors of traumatic brain injury: a prospective evaluation in a Jakarta University hospital. J Clin Neurosci.

[18] Kehoe A, Rennie S, Smith JE (2015). Glasgow Coma Scale is unreliable for the prediction of severe head injury in elderly trauma patients. Emerg Med J.

[19] Demetriades D, Kuncir E, Murray J, Velmahos GC, Rhee P, Chan L (2004). Mortality prediction of head Abbreviated Injury Score and Glasgow Coma Scale: analysis of 7,764 head injuries. J Am Coll Surg.

[20] Timmons SD, Bee T, Webb S, Diaz-Arrastia RR, Hesdorffer D (2011). Using the abbreviated injury severity and Glasgow Coma Scale scores to predict 2-week mortality after traumatic brain injury. J Trauma.

[21] Foreman BP, Caesar RR, Parks J, Madden C, Gentilello LM, Shafi S (2007). Usefulness of the abbreviated injury score and the injury severity score in comparison to the Glasgow Coma Scale in predicting outcome after traumatic brain injury. J Trauma.

[22] Airaksinen NK, Heinänen MT, Handolin LE (2019). The reliability of the ICD-AIS map in identifying serious road traffic injuries from the Helsinki Trauma Registry. Injury.

[23] Clark DE, Black AW, Skavdahl DH, Hallagan LD (2018). Open-access programs for injury categorization using ICD-9 or ICD-10. Inj Epidemiol.

[24] Haas B, Xiong W, Brennan-Barnes M, Gomez D, Nathens AB (2012). Overcoming barriers to population-based injury research: development and validation of an ICD10-to-AIS algorithm. Can J Surg.

[25] Loftis KL, Price JP, Gillich PJ, Cookman KJ, Brammer AL, St Germain T (2016). Development of an expert based ICD-9-CM and ICD-10-CM map to AIS 2005 update 2008. Traffic Inj Prev.

